# Chemical Compositions, Extraction Optimizations, and In Vitro Bioactivities of Flavonoids from Perilla Leaves (*Perillae folium*) by Microwave-Assisted Natural Deep Eutectic Solvents

**DOI:** 10.3390/antiox12010104

**Published:** 2022-12-31

**Authors:** Xianchao Shang, Manman Zhang, Jing Hu, Yuqin Zhang, Long Yang, Xin Hou

**Affiliations:** 1 College of Plant Protection and Agricultural Big-Data Research Center, Shandong Agricultural University, Taian 271018, China; 2 Citrus Research Institute, Southwest University, Chongqing 400712, China; 3 Weihai Academy of Agricultural Sciences, Weihai 264200, China

**Keywords:** *Perillae folium*, natural deep eutectic solvents, microwave-assisted extraction, chemical compositions, antimicrobial activity, response surface methodology

## Abstract

Natural deep eutectic solvents (NADESs) have been gradually applied to green extraction of active ingredients. In this study, microwave-assisted NADESs were applied to the extraction of flavonoid compounds from perilla leaves. Through comparative experiments, NADES-3 (choline chloride and malic acid at a molar ratio of 1:1) was found to have the highest extraction efficiency of total flavonoids, including apigenin 7-O-caffeoylglucoside, scutellarein 7-O-diglucuronide, luteolin 7-O-diglucuronide, and scutellarein 7-O-glucuronide by HPLC-MS. The following optimal extraction parameters were obtained based on response surface design: water content in NADES of 23%, extraction power of 410 W, extraction time of 31 min, and solid–liquid ratio of 75 mg/mL, leading to the extraction yield of total flavonoids of 72.54 mg/g. Additionally, the strong antimicrobial and antiallergic activity, inhibition of nitrosation, and antioxidant activity of total flavonoids by using NADESs were confirmed. This new extraction method provides a reference for the further exploration of NADES systems and may be widely used for the green extraction of natural active ingredients.

## 1. Introduction

Perilla (*Perilla frutescens*) is an annual herb belonging to Labiatae in the mint family [1,2]. Perilla resources are all over the world, mainly planted in Asian countries, such as China, India, South Korea, Vietnam, Thailand, and Japan, etc. [3,4]. Perilla leaves are rich in nutrients, and the crude protein content in the leaves is 27.8%, which is far more than the that in ordinary vegetable leaves [1,2,5]. In previous studies, eight different kinds of essential amino acids, a variety of trace mineral elements needed by the human body, α-linolenic acid, ω-3-polyunsaturated fatty acids, and ascorbic acid were identified and isolated from perilla leaves [6,7,8,9]. Due to these multiple substances, bioactive effects, including antimicrobial, antiallergy, antioxidation, anti-inflammation, and anticancer effects have been confirmed [6,8,10,11], which leads to its potential application value in functional foods.

Flavonoids, also known as plant flavonoids or bioflavonoids, are a kind of low-molecular-weight natural plant ingredient from fruits, vegetables, tea, wine, seeds, and plant roots [12,13]. As the secondary metabolites of plants, more than 4000 compounds belong to flavonoids, which have excellent bioactive effects and are widely used in biology, food, and medicine [12]. Perilla leaves have been proved to contain a large number of flavonoids [14,15]. Therefore, increasing the yield of flavonoid compounds and enhancing their compositions become the top priority. As reported, traditional extraction methods, such as organic solvents extraction supplemented by ultrasonication or microwaves, could improve extraction efficiency [2,8,16]. However, considering environmental protection, it is urgent that a new class of solvent should be found to replace organic solvent for extracting flavonoids from perilla leaves.

Natural deep eutectic solvents (NADESs), composed of low-cost biodegradable compounds with low toxicity, can replace traditional organic solvents as extractants [17,18,19,20]. NADESs have attracted extensive attention from researchers due to their good water solubility, degradability, biocompatibility, high selectivity, recyclability, high efficiency, and easy preparation [21,22,23,24]. At present, it has been reported that NADESs are used as the extractant in microwave extraction to extract the active target in plant resources [25,26,27]. For example, the phenolic acids [28] and tanshinones [29] were extracted from *Carthamus tinctorius* L. and *Salvia miltiorrhiza* bunge by using seven and six different NADESs, respectively.

Recently, a study was conducted on flavonoid extraction with NADESs from *Lycium barbarum* L. fruits, which initially suggested that NADESs were a potential extractant for flavonoid compounds [30]. Compared with the application of organic solvents (i.e., methanol and ethanol), NADESs applied in the extraction of flavonoids from perilla leaves have not been deeply studied. Based on the methods with NADESs, this study established an extraction method to apply microwave-assisted NADESs for the perilla leaf total-flavonoid extraction. The extraction ability of NADES systems with different components was observed, and the yields of total flavonoids were optimized using a single-factor experiment and a response surface design. The chemical compositions of total flavonoids extracted by NADESs were analyzed by high-performance liquid chromatography–mass spectrometry (HPLC-MS). To further prove the biological activity of total flavonoids, the antimicrobial activity, antiallergic activity, inhibition of nitrosation, and antioxidant activity were studied, respectively.

## 2. Materials and Methods

### 2.1. Materials and Chemicals

The fresh leaves of perilla (*Perillae folium*) were collected from Longquan city, Zhejiang province, China. All leaf samples were kept and used in the laboratory of Shandong Agricultural University (Taian, China). The leaves were freeze-dried in a freeze-dryer (FD-2C, Bilang Instrument Co., Ltd., Shanghai, China) at −50 °C for 72 h to ensure that the final mass remained constant. After drying, leaves were pulverized into powder and the appropriate powder of 60-mesh was selected to obtain perilla leaf flavonoids.

Choline chloride, D-(−)-Fructose, D-(+)-glucose, malic acid, citric acid, glycerol, ethylene glycol, and urea for NADES synthesis were all purchased from Aladdin Reagent Company (Shanghai, China). Acetonitrile and trifluoroacetic acid of HPLC grade were obtained from Shanghai Macklin Biochemical Co., Ltd. (Shanghai, China). The other reagents and chemicals used in this work were all of analytical grade.

### 2.2. Synthesis of NADESs

The synthesis of the NADESs was carried out according to the methods described in a previous study [24]. Component 1, as hydrogen bond acceptors (HBAs, component 1), were mixed with hydrogen bond donors (HBDs, component 2) at different molar ratios (Table 1), and then the mixed system was put into an oil bath at 80 °C for 2–3 h. NADESs were successfully obtained once the mixed system became a transparent homogeneous liquid.

### 2.3. Microwave-Assisted Extraction of Total Flavonoids from Perilla Leaves by Using Different NADESs (NADES-MAE)

The dried powder (100 mg) of perilla leaves was added into the 1 mL of NADES systems (water content in the NADES was 30%, *w*/*w*) in Table 1 and sufficiently stirred. Then, the mixture was placed in a microwave extraction instrument and kept for 20 min under the set microwave conditions at 300 W. The supernatant was completely collected after centrifugation for 10 min at 6000 r/min for the quantitative analysis.

### 2.4. Assessment of Total Flavonoids from Perilla Leaves

**Figure A1:**
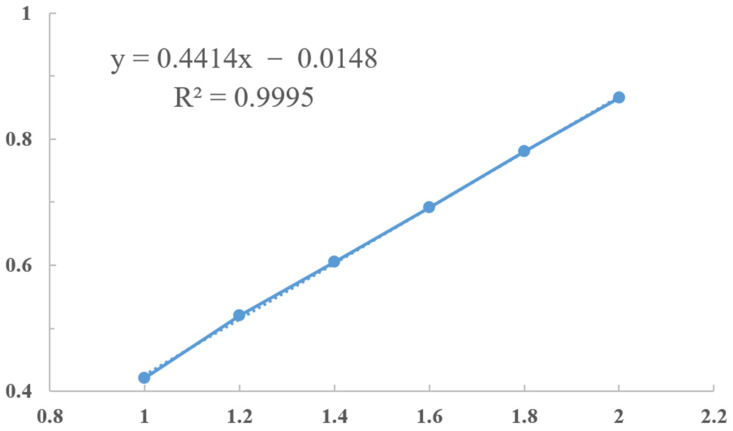
The standard curve of rutin.

The yields of total flavonoids from perilla leaves were determined by the color method of sodium nitrite–aluminum nitrate, which was expressed as milligrams of rutin equivalents per gram of perilla leaves (mg rutin equivalent/g perilla leaves, mg/g). The standard curve and formula of rutin are shown in Figure A1.

### 2.5. HPLC-MS Analysis

The perilla leaves total flavonoids derived from the optimized NADES extraction system was selected and their chemical compositions were identified by HPLC-MS using a liquid chromatography/mass spectrometry system (LC-MS200, Shanghai Jinghong Experimental Instrument and Equipment Co., Ltd., Shanghai, China) equipped with an Insertsil ODS-SP column (250 mm × 4.6 mm, 5 μm). Trifluoroacetic acid aqueous solution (5 mmol/L, 0.1% formic acid *v*/*v*) and acetonitrile were used as mobile phases A and B, respectively. The column temperature, flow rate, and injection volume were 35 °C, 0.3 mL/min, and 5 μL, respectively. The gradient elution method was as follows: 0–15 min, 80–50% A; 15–17 min, 50% A; 17–17.1 min, 50–80% A; 17.1–20 min, 80% A, stop. For the MS detection, the positive and negative ions of electron spray ionization (ESI) were simultaneously scanned. Other parameters of the mass spectrometer were as follows: ion source temperature of 400 °C, flow rate of atomizing gas of 1.5 mL/min, and interface voltage of the ion source of 4.5 kV.

### 2.6. Single-Factor Optimization Experiment of NADES-MAE

In order to effectively improve the yield of total flavonoids from perilla leaves by using NADES, the four key parameters in this study were optimized through single-factor experiments. The effects of water content in NADES (0, 10%, 20%, 30%, 40%, and 50%), extraction power (100, 200, 300, 400, 500, and 600 W), extraction time (10, 20, 30, 40, 50, and 60 min), and solid–liquid ratio (20, 40, 60, 80, 100, and 120 mg/mL) on extraction efficiency were investigated, respectively.

### 2.7. Response Surface Methodology Optimization of NADES-MAE and Separation of Perilla Leaf Extract

On the basis of single-factor optimization experiments, response surface methodology (RSM) was further used to optimize the optimal extraction parameters by Design-Expert Ver. 8.1.5 (Stat-Ease Inc., Minneapolis, MN, USA). Box–Behnken design (BBD) with four independent variables, including water content in the NADES (A), extraction power (B), extraction time (C), and the solid–liquid ratio (D), was completed to evaluate the interactions among them (Table A1). Rightfully, the yields of total flavonoids from perilla leaves were treated as the responses of the BBD. This prediction model was developed by multiple regression analysis, correlating the responses with independent factors. To more intuitively reflect the interactions between the various variables, three-dimensional (3D) response surface plots were obtained in this study.

The supernatants collected under optimal extraction conditions were loaded onto a macroporous resin column (1000 mm × 50 mm), which was sequentially flushed with 2000 mL of deionized water at a flow rate of 10 mL/min (remove the NADES firstly), and methanol (500 mL) at 20 mL/min. Eventually, the methanolic extract was completely concentrated, and the concentrate was dried with nitrogen and then stored at 4 °C for further biological activity analysis.

### 2.8. Antimicrobial Activity

The microbial strains used in this study included Gram-negative *Escherichia coli* PT 1362 (*E. coli*); Gram-positive *Staphylococcus aureus* PT 1923 (*S. aureus*) and *Bacillus subtilis* PT 521 (*B. subtilis*); and *Candida albicans* PT 239 (*C. albicans*) fungi. All strains were obtained from the College of Plant Protection of Shandong Agricultural University. Antimicrobial activities of total flavonoids were estimated from perilla leaves against *E. coli*, *S. aureus*, *B. subtilis*, and *C. albicans* using the disc diffusion method, according to the previous standards.

The bacterial suspensions (10^6^ CFU/mL, 100 μL) of *E. coli*, *S. aureus*, and *B. subtilis* were coated on nutrient agar (NA) medium, while for *C. albicans*, potato dextrose agar (PDA) medium plates were selected as solid medium. The sample of total flavonoids was diluted to 200 μg/mL with dimethyl sulfoxide (DMSO). Later, a sterile filter paper disc (with a diameter of 5 mm) saturated with the solution of total flavonoids was softly placed at the center of the solid medium. All of the plates were kept at 4 °C for 30 min, after which the fungus-cultured media were cultivated at 28 °C for 48 h and the bacterium-cultured media were cultivated at 37 °C for 36 h. After incubation, the diameter of the inhibition zone was measured, comparing it with the blank control (a sterile filter disc with DMSO) and positive control (a sterile filter disc with penicillin–streptomycin solution, 1000 U/mL). All experiments in different groups and strains were replicated three times.

### 2.9. Antiallergic Activity

Hyaluronidase is involved in type I allergic reactions, which are closely related to allergy and inflammation [31]. Therefore, the in vitro antiallergic activity of total flavonoids was determined using the Elson–Morgan method. Different concentrations (0.04, 0.08, 0.12, 0.24, 0.48, 0.96, and 1.92 mg/mL) of the total flavonoid solution were determined, and tea polyphenols were used as positive controls. The hyaluronidase inhibition rate was calculated according to the following formula:

Hyaluronidase inhibition rate (%) = [(A1 − A2) − (A3 − A4)]/(A1 − A2) × 100%

where A1 is the absorbance value of the positive control solution; A2 is the absorbance value of the positive control–blank solution; A3 is the absorbance value of the total flavonoid solution; and A4 is the absorbance value of the total flavonoid–blank solution.

### 2.10. Inhibition of Nitrosation

The effect of total flavonoids on scavenging nitrite was measured by employing a modified method in our laboratory. Briefly, 1 mL of sample (0.003, 0.006, 0.009, 0.030, 0.060, 0.090, and 0.120 mg/mL) was put into a 25 mL volumetric flask, and then 15 mL of sodium citrate hydrochloric acid buffer solution (pH of 3.0) and 5 mL of NaNO_2_ solution (5 mg/L) were added. After fully mixing, the total volume was fixed to the scale with distilled water, and the constant temperature was maintained at 37 °C for 1.5 h. Subsequently, 1 mL of reaction solution was sucked into a small test tube, mixed with 2 mL of p-aminobenzenesulfonate solution (0.4%, *m*/*v*) and 2 mL of N-1-naphthalene ethylenediamine hydrochloride solution (0.2%, *m*/*v*), and then shaken up and placed for 15 min. The absorbance value of the reaction solution was measured at the wavelength of 540 nm with a spectrophotometer. Vitamin C was selected as the positive control in order to intuitively display the scavenging capacity of total flavonoids. The scavenging rate of nitrite was calculated according to the following formula:

Nitrite scavenging rate (%) = (A0 − A1)/A0 × 100%

where A0 is the absorbance value of the blank solution, and A1 is the absorbance value of the total flavonoid solution.

### 2.11. Antioxidant Activity

#### 2.11.1. Determination of DPPH Free Radical Scavenging Capacity

The determination of the DPPH free radical (a very stable, nitrogen-centered free radical) scavenging rate is often used to evaluate the antioxidant capacity of various active substances in vitro [32]. According to the experimental methods from previous studies [27,32] and appropriately modified, 2.5 mL of DPPH ethanol solution (0.2 mmol/L) was added into 0.5 mL of each different concentration (50, 100, 150, 200, 250, and 300 μg/mL) of perilla leaf extract. After an airtight reaction under dark conditions for 40 min, the absorbance of the supernatant was measured at the wavelength of 517 nm. Butylated hydroxytoluene (BHT) and vitamin E were selected as the positive controls in this study. The scavenging rate of the DPPH free radical was calculated according to the following formula:

DPPH free radical scavenging rate (%) = (A0 − A1)/A0 × 100%

where A0 is the absorbance value of the blank solution, and A1 is the absorbance value of the total flavonoid solution.

#### 2.11.2. Determination of ABTS Free Radical Scavenging Capacity

First, 1 mL of perilla leaf extract solution at different concentrations (50, 100, 150, 200, 250, and 300 μg/mL) was added into 10 mL test tubes, and then 4 mL of ABTS^+^· working solution (OD_734_ = 7.0 ± 0.2) was fully mixed into the system. After ten minutes of reaction, the absorbance of the mixed solution was measured at the wavelength of 734 nm. Similarly, the ABTS free radical scavenging rate of BHT and vitamin E were used as positive controls in this study. The scavenging rate of the ABTS free radical was calculated according to the following formula:

ABTS free radical scavenging rate (%) = (A0 − A1)/A0 × 100%

where A0 is the absorbance value of the blank solution, and A1 is the absorbance value of the total flavonoid solution.

### 2.12. Statistical Analysis

All test data were measured in triplicate, and the results were expressed as means ± standard error. Data processing and figure drawings were supported by SPSS Statistics 20.0 (Chicago, IL, USA) and Origin 8.5 (Northampton, MA, USA), respectively. Differences between treatments were analyzed either by a Student’s *t*-test or ANOVA, followed by Tukey’s post hoc test. Different letters in the figures indicate a significant difference (*p* < 0.05).

## 3. Results and Discussions

### 3.1. Screening the Optimal NADES

**Figure 1:**
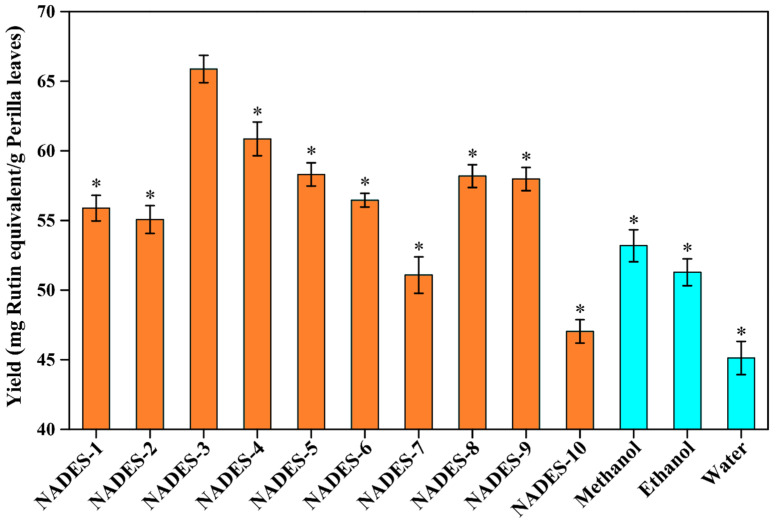
The extraction yields of total flavonoids from perilla leaves by using different types of NADESs. The preferred test conditions were as follows: water content in the NADES systems, 30% (*w*/*w*); extraction power, 300 W; extraction time, 20 min; solid–liquid ratio, 100 mg/mL. The * indicates that yields of total flavonoids were significantly different from NADES-3 (*p* < 0.05).

Based on the quantitative analysis (Figure 1), significant variability was observed in the efficiency of extracting total flavonoids from perilla leaves by using different NADESs, ranging from 47.03 mg/g to 65.87 mg/g. This might be caused by different NADESs with various special physical and chemical properties [18,19]. As displayed in Figure 1, the extraction yields of the NADES-2, NADES-7, and NADES-10 groups were only 55.07 mg/g, 51.08 mg/g, and 47.03 mg/g, respectively. The maximum yield was 65.87 ± 1.00 mg/g from NADES-3, which increased by 17.10% compared with that of the NADES-10 group. Compared with conventional extraction solvents, the extraction yield of DES-3 was significantly higher than those of methanol (53.19 ± 1.15 mg/g), ethanol (51.28 ± 0.97 mg/g), and water (45.12 ± 1.19 mg/g), suggesting the higher efficiency of NADESs in the extraction of total flavonoids from perilla leaves. NADES-4 (60.86 mg/g) was the second, followed by NADES-5 (58.31 mg/g), NADES-8 (58.19 mg/g), NADES-9 (57.98 mg/g), NADES-6 (56.45 mg/g), and NADES-1 (55.89 mg/g). Generally, the compositions (types of HBA and HBD) of the NADESs can affect their extraction capacities for bioactive compounds from plants [13,20]. The high viscosity of the NADESs (NADES-1, NADES-2, NADES-7, NADES-8, NADES-9, and NADES-10) hampered mass transfer and diffusion of flavonoids, leading to inferior extraction yields [18,21]. The alcohol/ChCl-NADESs (NADES-5 and NADES-6) resulted in insufficient chloride anions in the system and weakened the interaction between flavonoids and NADESs, which reduced the extraction yields [18,21,23]. NADES-3 and NADES-4 can have better interactions with the less-polar flavonoids, which result in the higher extraction yields. In addition, due to the difference in HBDs between NADES-3 and NADES-4, their intermolecular interactions are different, which might make NADES-3 show the best extraction efficiency in this study. According to both previous studies and our results [23], appropriate screening of the kinds of NADESs could optimize the yields of total flavonoids from perilla leaves. Hence, NADES-3 (composed of choline chloride and malic acid, with a 1:1 molar ratio) was finally selected as the optimal solvent for the further extraction and determination.

### 3.2. HPLC-MS Analysis of Flavonoids from Perilla Leaves

**Figure 2:**
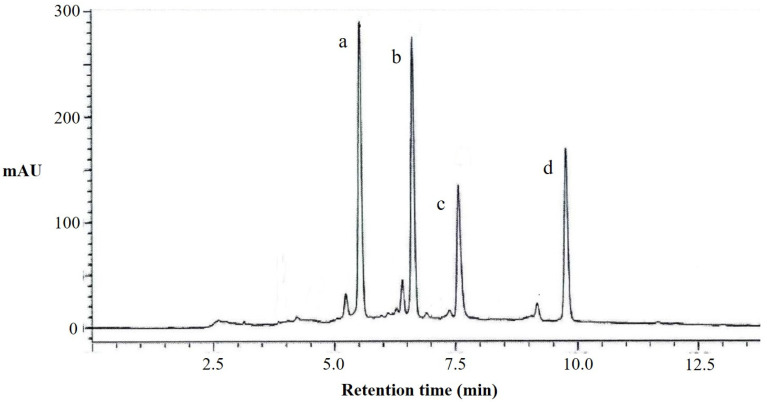
HPLC chromatograms at 330 nm of flavonoids from perilla leaves. a, apigenin 7-O-caffeoylglucoside; b, scutellarein 7-O-diglucuronide; c, luteolin 7-O-diglucuronide; and d, scutellarein 7-O-glucuronide.

According to the results of the liquid chromatography (Figure 2) and mass spectrometry analysis (Table A2) of the total flavonoids extracted from perilla leaves, we observed that it contains four kinds of flavonoid compounds (apigenin 7-O-caffeoylglucoside, scutellarein 7-O-diglucuronide, luteolin 7-O-diglucuronide, and scutellarein 7-O-glucuronide). The peak a in liquid chromatography was apigenin 7-O-caffeoylglucoside, with a molecular weight of 595, which is formed by the substitution of apigenin in position seven with caffeoylglucoside. The peaks b, c, and d were scutellarein 7-O-diglucuronide, luteolin 7-O-diglucuronide, and scutellarein 7-O-glucuronide, respectively. It can be seen that the flavonoid compounds in perilla leaves are mainly different compounds formed by the substitutions of baicalein, apigenin, and luteolin in position seven with different glucosides, determining that their structures are similar.

### 3.3. Single-Factor Experiments of NADES-MAE

**Figure 3:**
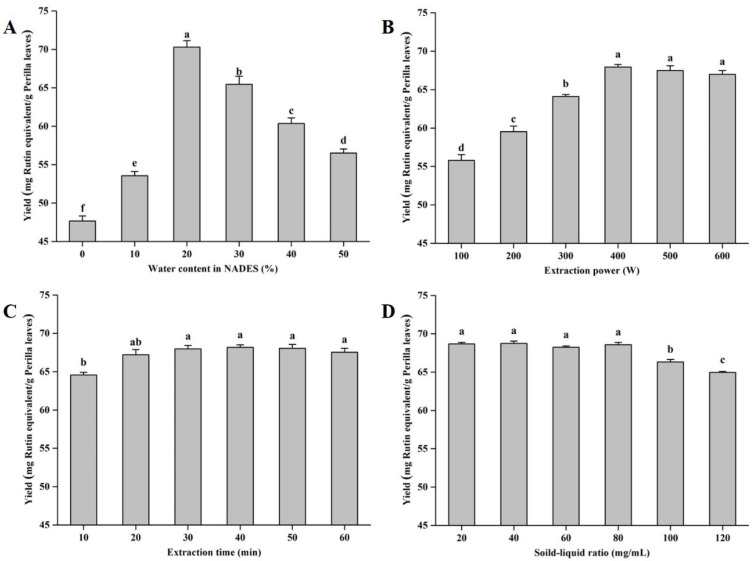
The extraction yields of total flavonoids from perilla leaves with different influencing factors. Invariant extraction conditions: 300 W, 20 min, and 100 mg/mL (**A**); 30%, 20 min, and 100 mg/mL (**B**); 30%, 300 W, and 100 mg/mL (**C**); and 30%, 300 W, and 20 min (**D**). Data sharing the different letters were significantly different (*p* < 0.05).

Hydrogen bonding is the core of the action of NADESs, and the water content in the system will affect the viscosity of the NADES, which will have a significant impact on its extraction efficiency [28,30]. With the increase in the water content in the NADES, the extraction yields first increased and then decreased (Figure 3A). This might be because, with the initial increase in the water ratio, the hydrogen bond effect of the NADES is enhanced, and more flavonoid monomers are depolymerized [27]. With the excessive addition of water, a certain threshold is broken, resulting in a decrease in the extraction capacity of the NADES [24]. Based on the results of single-factor experiments, the optimal water content in NADESs was determined to be 20% (*w*/*w*). Additionally, the effects of extraction power, extraction time, and on the yields of total flavonoid compounds from perilla leaves were evaluated. As displayed in Figure 3B–D, the optimal values of extraction power, extraction time, and solid–liquid ratio were 400 W, 30 min, and 80 mg/mL, respectively.

### 3.4. NADES-MAE Optimization by Response Surface Design

For the results of single-factor experiments, the Box–Behnken design (one response surface method) experiment with four factors and three levels was used in this study. In this study, the water content in the NADES (A), microwave power (B), extraction time (C), and solid–liquid ratio (D) were defined as independent variables, and the yields of the total flavonoids were treated as response values. The quadratic polynomial fitting was performed on the data in Table 2. The second-order regression equation of the prediction model was as follows:

Yield = 72.09 + 1.57 × A + 0.1767 × B + 0.1383 × C − 0.5275 × D + 0.0825 × AB − 0.0175 × AC − 0.1425 × AD − 0.0075 × BC + 0.12 × BD + 0.015 × BD − 2.41 × A^2^ − 0.9142 × B^2^ − 1.07 × C^2^ − 1.16 × D^2^

The water content in the NADES (A), microwave power (B), extraction time (C), and solid–liquid ratio (D) in the equation were all treated by dimensional linear coding in the design. Thus, the absolute value of each coefficient in the equation directly reflected the degree of influence of each factor on the values of the extraction yields of the total flavonoids, and the positive and negative coefficients reflected the directions of the influences.

The mathematical prediction model of the yields of total flavonoids was also analyzed by variance (Table 3). The *p*-value of the regression model was <0.0001, indicating that this model had very high significance (*p* < 0.01), and the *p*-value of lack of fit was 0.4393, which proved that the lack of fit was not significant (*p* > 0.05). In addition, the adjusted R^2^ was 0.9199, indicating that the experimental design was reasonable, and the prediction model could accurately reveal the relationships between variables and extraction yields. The significance analysis of the equation showed that the primary items (A and D) and the secondary items (A^2^, B^2^, C^2^, and D^2^) had very significant impacts on the yields of total flavonoids from perilla leaves (*p* < 0.01), while B and C and the interactive items AB, AC, AD, BC, BD, and CD had no significant impacts (*p* > 0.05). According to the F value of the variance, the orders of the four factors affecting the yields of total flavonoids were: water content in NADES (129.12) > solid–liquid ratio (14.59) > extraction power (1.64) > extraction time (1.00).

**Figure 4:**
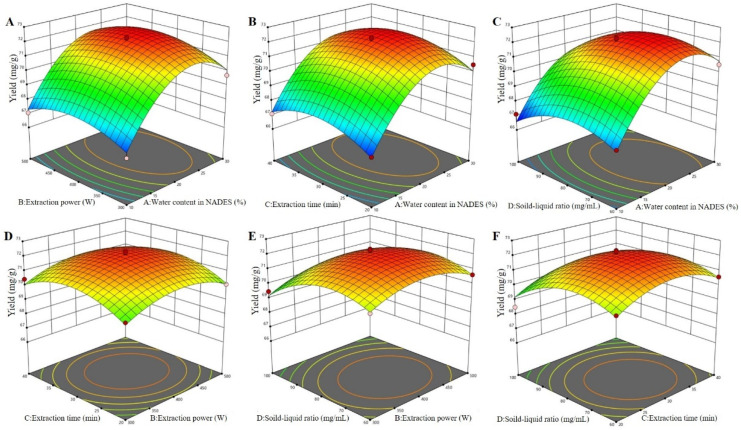
The 3D surfaces of the Box–Behnken design for NADES-MAE optimization. Water content in the NADES and extraction power (**A**); water content in the NADES and extraction time (**B**); water content in the NADES and solid–liquid ratio (**C**); extraction power and extraction time (**D**); extraction power and solid–liquid ratio (**E**); and extraction time and solid–liquid ratio (**F**).

By analyzing the 3D response surfaces (Figure 4), the greater the slope of the surface, the more significant the interaction of the two factors on the extraction effect [27]. As shown in Figure 4, the surface of interaction between water content in the NADES and the solid–liquid ratio was steeper than that of the interaction between other factors. This indicated that the interaction between water content in the NADES and the solid–liquid ratio had a greater impact on the yields of total flavonoids from perilla leaves than that of interactions between other factors, which was consistent with the analysis of variance. Furthermore, the shape of the contour line reflected the strength of the interaction, and the ellipse indicated that the interaction between the two factors was significant, while the circle indicated the opposite. It was observed that the yield of total flavonoids increased rapidly as water content increased from 10% to 23% but decreased with higher percentages. As exhibited in Figure 4A–C, the maximum yield of total flavonoids was observed when water content was 23%. This may be due to the decrease in HBA basicity and increase in polarizability/dipolarity with the addition of water [17,19,21]. As reported by Mohammad et al. [30], water content alters the viscosity and polarity of the NADESs, allowing one to tailor the properties of NADESs and make them ideal extraction media for the active compounds in the presence of suitable water content. It was also noted that the viscosities of the DESs were highly sensitive to the solid–liquid ratio. The positive effect of the solid–liquid ratio on the extraction of total flavonoids seemed to be the result of the solvent influence on diffusion and solubility rates. The maximum yield reached when the solid–liquid ratio was less than 75 mg/mL (Figure 4). All the above results verified the rationality of this model. 

Based on the analysis of the Design Expert software, the optimal technological conditions for the extraction of total flavonoids from perilla leaves were obtained as follows: water content in the NADES of 23.35%, extraction power of 409.58 W, extraction time of 30.60 min, and solid–liquid ratio of 75.16 mg/mL. The theoretical yield of total flavonoids extracted under this condition was 72.43 mg/g. Considering the actual production, the modified optimal process conditions were obtained as follows: water content in the NADES of 23%, extraction power of 410 W, extraction time of 31 min, and solid–liquid ratio of 75 mg/mL. Thus, the verified extraction yield of total flavonoids was 72.54 mg/g, which was very close to the predicted value, indicating that this model was actual and reliable.

### 3.5. Antimicrobial Activity of Total Flavonoids from Perilla Leaves

The antimicrobial activities of perilla leaf extracts on bacteria and fungi have been investigated in many studies [3,5]. To evaluate the availability of total flavonoids from perilla leaves by using NADES groups, antimicrobial activities against *E. coli*, *S. aureus*, *B. subtilis*, and *C. albicans* were carried out in this study, respectively. The results of the inhibition zone diameter are summarized in Table 4. As expected, the positive control had the strongest inhibition effect on all microbial strains and no obvious inhibition zone was observed on the medium with only the DMSO solution. The total flavonoid compounds showed weakest inhibition effects toward *E. coli* (7.48 ± 0.80 mm), compared with *S. aureus* (15.47 ± 0.35 mm), *B. subtilis* (14.92 ± 0.62 mm), and *C. albicans* (11.33 ± 0.31 mm). According to the above results, *S. aureus* and *B. subtilis* were the two strains most susceptible to perilla leaf total flavonoids extracted by NADESs, while *C. albicans* was medium and *E. coli* presented the lowest susceptibility. The differences in bacteriostatic activity might be attributed to the effective chemical compositions, such as apigenin 7-O-caffeoylglucoside and scutellarein 7-O-diglucuronide in Section 3.2, which would be worth further study in antimicrobial applications of natural products extracted by NADESs.

### 3.6. Antiallergic Activity of Total Flavonoids from Perilla Leaves

The total flavonoids from perilla leaves with different concentrations showed significant differences in the inhibition on hyaluronidase (Table A3). With the increase in total flavonoid concentrations, the inhibition rate of hyaluronidase increased rapidly. The inhibition rate of hyaluronidase reached 87.89% at 0.96 mg/mL. In the same way, the inhibition rate of tea polyphenols increased continuously at 0.04–0.12 mg/mL, and then the change trend of its inhibition rate tended to be flat with the continuous increase in the concentration. At the highest concentration (1.92 mg/mL), the inhibition rate of total flavonoids (90.44%) was far greater than that of tea polyphenols (74.11%).

### 3.7. Inhibition of Nitrosation by Total Flavonoids from Perilla Leaves

In human stomach organs, nitrite and amine substances can produce a strong carcinogen nitrosamine, which also leads to the oxidative destruction of vitamin A in the intestine and interferes with the transformation of carotene to vitamin A [33,34]. Therefore, it was of great significance to study the nitrite scavenging capacity of total flavonoids from perilla leaves by using NADESs. As shown in Table A4, perilla leaf total flavonoids had a certain scavenging capacity for nitrite. With the increase in total flavonoid concentrations, the scavenging rate gradually increased. When the concentration reached above 0.009 mg/mL, there was a higher nitrite scavenging rate (>10%). The scavenging rate of total flavonoids at 0.120 mg/mL was 64.58%, which was equivalent to the effect of vitamin C at 0.030 mg/mL.

### 3.8. Antioxidant Activity of Total Flavonoids from Perilla Leaves

**Figure 5:**
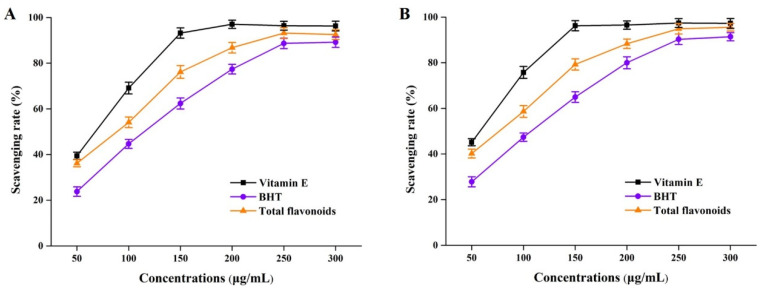
DPPH free radical (**A**) and DPPH free radical (**B**) scavenging rates of total flavonoid compounds in vitro. BHT, butylated hydroxytoluene.

It has been widely reported that flavonoids identified from Perilla frutescens organic solvent extracts played a critical role in antioxidative effects [6,7]. In this study, total flavonoid (derived from NADES groups) solutions of different concentrations were subjected to DPPH and ABTS assays. As the results presented in Figure 5 show, the DPPH and ABTS free radical scavenging experiments showed that total flavonoids from perilla leaves by using NADESs had better antioxidant properties. By comparing the scavenging rates under the same action concentrations, we found that the antioxidant capacity of perilla leaf total flavonoids was stronger than that of BHT, but much lower than that of vitamin E. These above phenomena were probably due to the comprehensive effects of total flavonoid compounds, such as the flavone substances detected by HPLC-MS in Section 3.2.

## 4. Conclusions

This study applied microwave-assisted NADES pretreatment to enhance the yields of total flavonoid compounds from perilla leaves. Yields of total flavonoids were distinctly increased by NADESs, and the best result was derived from NADES-3 synthesized by choline chloride and malic acid (molar ratio 1:1). Based on the response surface design, the main extraction parameters were optimized as follows: water content in the NADES of 23%, extraction power of 410 W, extraction time of 31 min, and solid–liquid ratio of 75 mg/mL, whose extraction yield was 72.54 mg/g. Moreover, the main components of perilla leaf total flavonoids were preliminarily determined by HPLC-MS, such as apigenin 7-O-caffeoylglucoside, scutellarein 7-O-diglucuronide, luteolin 7-O-diglucuronide, and scutellarein 7-O-glucuronide. Meaningfully, the total flavonoids proved to have antimicrobial activity toward some bacteria and fungi, especially the *S. aureus* and *B. subtilis* groups. Total flavonoids of hyaluronidase inhibition rate and nitrite, DPPH free radical, and ABTS free radical scavenging rates were tested: the biological activities of perilla leaf total flavonoids from NADES groups were extremely excellent, including antiallergic activity, inhibition of nitrosation, and antioxidant activity.

This work confirmed the effective effect of NADES-MAE and provided a theoretical basis for the food industry, especially the ones related to natural plant sources. Undoubtedly, NADESs have the potential for further utilization, with the goal of green and sustainable development in the food industry.

## Figures and Tables

**Table 1:** Preparation of different NADESs.

Solvent No.	NADESs Compositions		Molar Ratio
	Component 1	Component 2	
NADES-1	Choline chloride	D-(+)-Glucose	1:1
NADES-2	Choline chloride	D-(−)-Fructose	1:1
NADES-3	Choline chloride	Malic acid	1:1
NADES-4	Choline chloride	Citric acid	1:1
NADES-5	Choline chloride	Glycerol	1:1
NADES-6	Choline chloride	Ethylene glycol	1:1
NADES-7	Choline chloride	Urea	1:1
NADES-8	D-(−)-Fructose	Malic acid	1:1
NADES-9	D-(+)-Glucose	Malic acid	1:1
NADES-10	D-(+)-Glucose	Citric acid	1:1

**Table 2:** The results of Box–Behnken design for NADES-MAE optimization.

Run	Variables				Yields (mg/g)
	A (%)	B (W)	C (min)	D (mg/mL)	
1	20	400	30	80	72.28 ± 0.67
2	20	500	40	80	70.57 ± 0.58
3	10	400	40	80	67.08 ± 0.37
4	10	500	30	80	67.06 ± 0.42
5	30	400	20	80	70.49 ± 0.67
6	20	500	30	100	70.17 ± 0.55
7	20	400	30	80	72.25 ± 0.71
8	20	300	20	80	69.93 ± 0.44
9	20	400	30	80	71.31 ± 0.47
10	20	400	20	60	70.38 ± 0.62
11	30	400	30	60	70.53 ± 0.56
12	20	300	30	60	70.41 ± 0.52
13	20	400	30	80	72.27 ± 0.79
14	30	300	30	80	69.71 ± 0.34
15	20	400	40	60	70.58 ± 0.42
16	10	400	20	80	66.94 ± 0.42
17	30	400	40	80	70.56 ± 0.43
18	20	300	30	100	69.48 ± 0.51
19	20	300	40	80	70.44 ± 0.39
20	10	400	30	60	67.46 ± 0.40
21	20	500	20	80	70.09 ± 0.47
22	20	400	40	100	68.77 ± 0.38
23	20	400	20	100	68.51 ± 0.36
24	10	400	30	100	67.11 ± 0.42
25	10	300	30	80	66.76 ± 0.19
26	20	400	30	80	72.34 ± 0.25
27	30	500	30	80	70.34 ± 0.37
28	20	500	30	60	70.62 ± 0.24
29	30	400	30	100	69.61 ± 0.29

A, water content in NADES; B, extraction power (W); C, extraction time (min); D, solid–liquid ratio (mg/mL).

**Table 3:** ANOVA of the regression model for NADES-MAE optimization.

Source	Sum of Squares	DF	Mean Square	*F* Value	*p*-Value
Model	76.80	14	5.49	23.97	<0.0001
A	29.55	1	29.55	129.12	<0.0001
B	0.3745	1	0.3745	1.64	0.2216
C	0.2296	1	0.2296	1.00	0.3335
D	3.34	1	3.34	14.59	0.0019
AB	0.0272	1	0.0272	0.1190	0.7353
AC	0.0012	1	0.0012	0.0054	0.9427
AD	0.0812	1	0.0812	0.3549	0.5608
BC	0.0002	1	0.0002	0.0010	0.9754
BD	0.0576	1	0.0576	0.2517	0.6237
CD	0.0009	1	0.0009	0.0039	0.9509
A^2^	37.53	1	37.53	164.01	<0.0001
B^2^	5.42	1	5.42	23.69	0.0002
C^2^	7.41	1	7.41	32.40	<0.0001
D^2^	8.70	1	8.70	38.00	<0.0001
Residual	3.20	14	0.2288		
Lack of Fit	2.44	10	0.2439	1.28	0.4393
Pure error	0.7650	4	0.1913		
Cor total	80.00	28			

**Table 4:** Diameter of inhibition zone of total flavonoids from perilla leaves.

Samples	Diameter of Inhibition Zone (mm)			
	*E. coli*	*S. aureus*	*B. subtilis*	*C. albicans*
Positive control	15.04 ± 0.66	29.60 ± 0.44	32.51 ± 0.74	20.52 ± 0.34
Solvent control	-	-	-	-
Total flavonoids	7.48 ± 0.80	15.47 ± 0.35	14.92 ± 0.62	11.33 ± 0.31

## Data Availability

Not applicable.

## Appendix A

**Table A1:** Independent variables and levels for Box-Behnken design.

Variables	Symbols	Coded Levels		
		−1	0	1
Water content in NADES (%)	A	10	20	30
Extraction power (W)	A	300	400	500
Extraction time (min)	B	20	30	40
Solid–liquid ratio (mg/mL)	C	60	80	100

**Table A2:** Identification of flavonoid compounds from perilla leaves by HPLC-MS.

Peak No.	Retention Time (min)	DAD-HPLC λ_max_	Mass Mode^+^	Flavonoid Compounds
a	5.62	329	595	Apigenin 7-O-caffeoylglucoside
b	6.67	334	287–639	Scutellarein 7-O-diglucuronide
c	7.57	348	287–639	Luteolin 7-O-diglucuronide
d	9.79	334	287–463	Scutellarein 7-O-glucuronide

**Table A3:** The inhibition rate of allergic reactions with total flavonoids from perilla leaves.

Concentrations (mg/mL)	Hyaluronidase Inhibition Rate (%)	
	Total Flavonoids	Tea Polyphenols
0.04	48.15%	44.07%
0.08	55.34%	66.10%
0.12	67.06%	70.43%
0.24	73.91%	72.03%
0.48	81.02%	73.21%
0.96	87.89%	73.06%
1.92	90.44%	74.11%

**Table A4:** The capacity of scavenging nitrite with total flavonoids from perilla leaves.

Concentrations (mg/mL)	Nitrite Scavenging Rate (%)	
	Total Flavonoids	Vitamin C
0.003	1.98%	35.57%
0.006	5.68%	48.71%
0.009	9.19%	53.69%
0.030	15.67%	65.58%
0.060	22.36%	73.25%
0.090	45.92%	80.18%
0.120	64.58%	85.84%
